# Efficacy of Terpenoid in Attenuating Aortic Atherosclerosis in Apolipoprotein-E Deficient Mice: A Meta-Analysis of Animal Studies

**DOI:** 10.1155/2019/2931831

**Published:** 2019-07-17

**Authors:** Han Liu, Yang Zhang, Siqiao Sun, Shuai Wang

**Affiliations:** ^1^Department of Respiration, The First Hospital of Jilin University, Changchun, Jilin, China; ^2^Department of Vascular Surgery, The First Hospital of Jilin University, Changchun, Jilin, China

## Abstract

**Background:**

The apolipoprotein E knockout (ApoE -/-) mouse model is well established for the study of terpenoids in the prevention of atherosclerosis. Studies investigating the clinical benefit of terpenoids in humans are scarce. This systematic review and meta-analysis evaluated the effects of terpenoid administration on atherosclerotic lesion area in ApoE -/- mice.

**Methods:**

A comprehensive literature search using PubMed, Embase, and the Cochrane Library databases was performed to identify studies that assessed the effects of terpenoids on atherosclerosis in ApoE -/- mice. The primary outcome was atherosclerotic lesion area, and study quality was estimated using SYRCLE's risk of bias tool.

**Results:**

The meta-analysis included 25 studies. Overall, terpenoids significantly reduced atherosclerotic lesion area when compared to vehicle control (*P*<0.00001; SMD: -0.55; 95% CI: -0.72, -0.39). In terpenoid type and dose subgroup analyses, sesquiterpenoid (*P*=0.002; SMD -0.93; 95% CI: -1.52, -0.34), diterpenoid (*P*=0.01; SMD: -0.30; 95% CI: -0.54, -0.06), triterpenoid (*P*<0.00001; SMD: -0.66; 95% CI: -0.94, -0.39), tetraterpenoid (*P*<0.0001; SMD: -1.81; 95% CI: -2.70, -0.91), low dose (*P*=0.0001; SMD: -0.51; 95% CI: -0.76, -0.25), medium dose (*P<*0.0001; SMD: -0.48; 95% CI: -0.72, -0.24), and high dose (*P*=0.002; SMD: -1.07; 95% CI: -1.74, -0.40) significantly decreased atherosclerotic lesion area when compared to vehicle control. PROSPERO register number is CRD42019121176.

**Conclusion:**

Sesquiterpenoid, diterpenoid, triterpenoid, and tetraterpenoid have potential as antiatherosclerotic agents with a wide range of doses. This systematic review provides a reference for research programs aimed at the development of terpenoid-based clinical drugs.

## 1. Introduction

Atherosclerosis is the main cause of cardiovascular disease, which is the leading cause of death globally [1, 2]. Dyslipidemia and oxidative stress are relevant to the pathogenesis of atherosclerosis [3, 4]. Therefore, statin-based lipid-modifying therapies, such as atorvastatin and rosuvastatin, are effective for lowering blood cholesterol levels and providing clinical benefits in patients with cardiovascular disease. However, the morbidity and mortality associated with atherosclerosis remain high [5], and there is an urgent unmet clinical need for novel prevention and treatment strategies [6].

In recent years, studies have shown that natural compounds, such as flavonoids, alkaloids, and terpenoids, attenuate atherosclerosis [7–9]. Terpenoids are a large and diverse class of naturally occurring organic chemicals that are similar to terpenes. Most terpenoids are multicyclic structures with oxygen-containing functional groups. Furthermore, terpenoids have a wide range of pharmacological effects, including antitumor, anti-inflammatory, antiatherosclerotic, and antimalarial activities [10–12]. The majority of studies on the antiatherosclerotic effects of terpenoids have focused on paclitaxel, [13, 14] which is a natural diterpene, and consensus on the antiatherosclerotic effects of other terpenoids has not been reached.

Studies in animals allow for initial investigations on the safety and efficacy of new interventions and provide an important link between basic research and clinical trials. The apolipoprotein E knockout (ApoE -/-) mouse model spontaneously develops atherosclerotic plaques and is commonly used to mimic the pathophysiological process of atherosclerosis in humans [15, 16]. The present systematic review and meta-analysis evaluated the effects of terpenoid administration on atherosclerotic lesion area in ApoE -/- mice, in an effort to understand the clinical potential of terpenoids as antiatherosclerotic agents.

## 2. Materials and Methods

### 2.1. Reporting Standards

This systematic review complies with the Preferred Reporting Items for Systematic Reviews and Meta-Analyses (PRISMA) statement. The systematic review protocol was prepared using the SYRCLE format for animal intervention studies [17, 18].

### 2.2. Search Strategy

An experienced information specialist (HL) searched the PubMed, Embase, and Cochrane Library databases from January 2001 to December 2018 using the keywords: “atherosclerosis,” “atherogenesis,” “apolipoproteins e,” “apoe,” “mice,” and “terpenoid” and the following search strategies: (atherosclerosis OR atherogenesis) AND (“apolipoprotein*∗*e” OR apoe) AND (mice OR mouse) AND (terpenoid OR hemiterpenoid OR monoterpenoid OR sesquiterpenoid OR diterpenoid OR sesterterpenoid OR triterpenoid OR tetraterpenoid OR polyterpenoid). The reference lists of included and review articles were manually searched to identify additional relevant studies. The search was performed on December 10, 2018, and was restricted to articles published in the English language.

### 2.3. Inclusion and Exclusion Criteria

Inclusion criteria are as follows: (1) study design: original research; (2) animal model: ApoE -/- mice; (3) disease model: atherosclerosis; (4) intervention: terpenoids. Exclusion criteria are as follows: (1) case reports, conference abstracts, review articles, and editorials, (2) missing data, or (3) overlapping or duplicate datasets.

### 2.4. Study Selection

Two reviewers (YZ and SS) independently examined the titles and abstracts of the articles identified by the literature search to select eligible studies. The full text of potentially relevant articles was retrieved and independently examined by two reviewers (YZ and SW) to determine whether these studies met the inclusion criteria. Disagreements on study selection were resolved by discussion and consensus.

### 2.5. Data Extraction

Two reviewers (HL and SW) independently extracted data from eligible studies, including the first author's name, publication year, age of mice, gender, diet, terpenoid dose, duration and route of treatment, control and treatment group sample sizes, location of the atherosclerotic lesion, stain used to assess the atherosclerotic lesion, and atherosclerotic lesion area. Data that were presented graphically in the original publications were extracted using Adobe Photoshop 7.0.

The primary outcome was atherosclerotic lesion area measured as a percentage or a numerical value.

Disagreements on data extraction were resolved by discussion and consensus.

### 2.6. Quality Assessment

Two investigators (SS and SW) independently assessed the quality of the included studies using SYRCLE's risk of bias tool, which contains domains evaluating sequence generation, baseline characteristics, allocation concealment, random housing, blinding, random and selective outcome assessments, incomplete outcomes data, and other sources of bias [44]. Publication bias was detected by visual inspection of funnel plots.

Disagreements on quality assessment were resolved by discussion and consensus.

### 2.7. Data Synthesis and Statistical Analysis

Statistical analyses were performed using Review Manager (RevMan Version 5.3 for Windows Copenhagen: The Nordic Cochrane Centre, The Cochrane Collaboration, 2014). Standardized mean differences (SMD) with 95% confidence intervals (CI) were calculated to reflect the effects of terpenoids or vehicle control on atherosclerotic lesion area. A random-effects model was used to pool studies. Heterogeneity was determined as moderate (*I*^*2*^ ≥ 30%) or high (*I*^*2*^ ≥ 50%) using the inconsistency index.

Multiple independent groups in a study (e.g., different terpenoid doses) were considered separate datasets. In eleven studies [19, 20, 24, 26, 28, 33, 34, 38–40, 42], multiple groups that tested different terpenoid doses were compared to a single control group. In order to avoid an artificial increase in sample size in the pooled analysis, the number of animals in the control group for each study was divided by the number of comparator groups.

Subanalyses were conducted to investigate the effects of sesquiterpenoid, diterpenoid, triterpenoid, and tetraterpenoid on the atherosclerotic lesion area.

Sensitivity analyses were conducted to determine whether the findings were robust.* P*<0.05 was considered statistically significant.

## 3. Results

### 3.1. Study Selection

**Figure 1:**
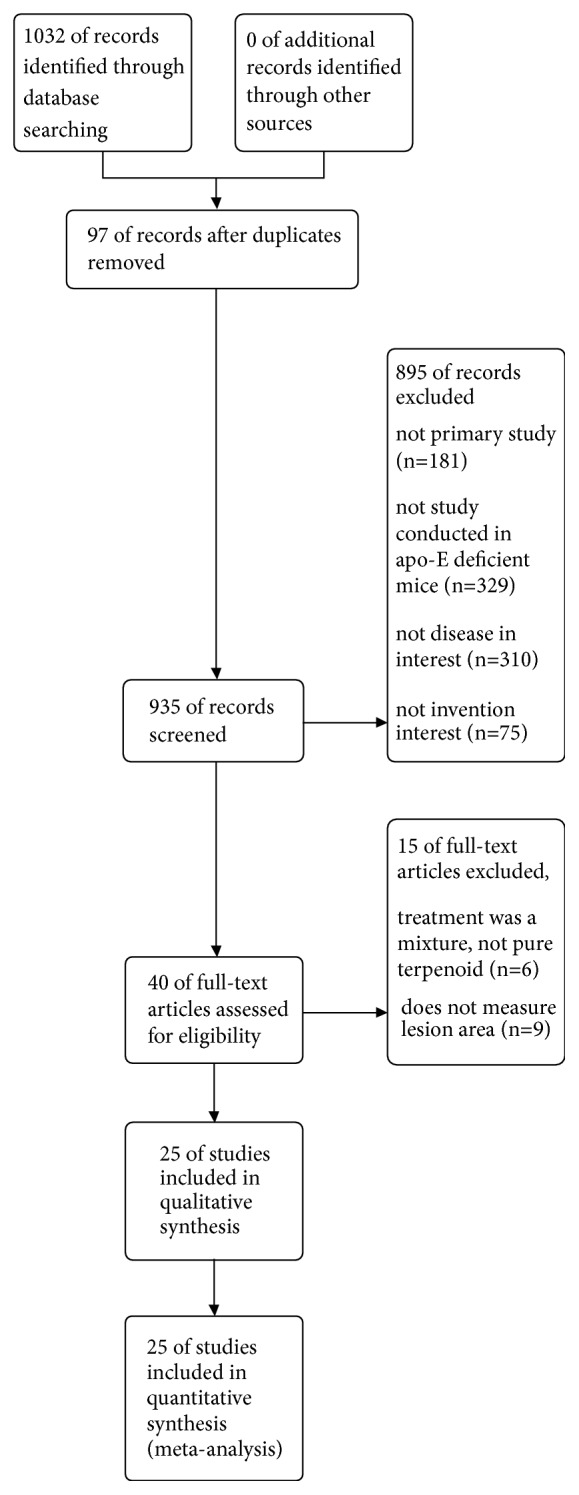
Flow diagram of the study identification and selection process.

The search identified 1,032 articles. Titles and abstracts were screened, and 40 studies were considered potentially eligible for inclusion. After evaluating full-text articles, nine studies were excluded, because outcomes data were not reported [45–53], and six studies were excluded, because multiple interventions were assessed [54–59]. Finally, 25 studies were included in the present meta-analysis [19–43] (Figure 1).

### 3.2. Study Characteristics

The characteristics of the 25 included studies are described in Table 1. These studies provided 59 datasets and involved 707 animals.

Three studies used sesquiterpenoid as the intervention [19–21], nine studies used diterpenoid as the intervention [22–30], ten studies used triterpenoid as the intervention [31–40], and three studies used tetraterpenoid as the intervention [41–43].

Two studies used female animals [21, 41], 21 studies used male animals [20, 22–35, 37–40, 42, 43], and the gender of the animals was not reported in two studies [19, 36].

Mice received normal chow diet in three studies [26, 41, 43], a high-cholesterol diet in two studies [27, 28], and a high-fat diet in 20 studies [19–25, 29–40, 42].

Terpenoid administration was initiated in 4-week-old mice in one study [19], in 6-week-old mice in seven studies [24, 26–28, 34, 36, 43], in 8-week-old mice in ten studies [21–23, 29, 30, 32, 33, 37, 38, 42], in 9-week-old mice in one study [35], in 10-week-old mice in two studies [25, 40], and in 12-week-old mice in four studies [20, 31, 39, 41].

The duration of terpenoid treatment varied from four weeks to 24 weeks.

Route of administration of terpenoid treatment was in drinking water in one study [38] and in the chow in seven studies [32, 34, 37, 39, 41–43], via an intragastric route in ten studies [21–29, 31] and via intraperitoneal injection in seven studies [19, 20, 30, 33, 35, 36, 40].

Terpenoid doses varied among different studies. It mainly ranged from 1 to 100 mg/kg/d. In addition, 500 and 2000 mg/kg/d were used in two studies [41, 43].

All studies reported an aortic-root or -sinus lesion area. Furthermore, ten studies [20, 25, 27, 30, 33, 35, 37, 41–43] reported cross-sectional aortic lesion area, five studies [19, 31, 36, 38, 39] reported longitudinal aortic lesion area (Table 1), and ten studies [21–24, 26, 28, 29, 32, 34, 40] reported both cross-sectional and longitudinal aortic lesion areas.

### 3.3. Quality Assessment

**Figure 2:**
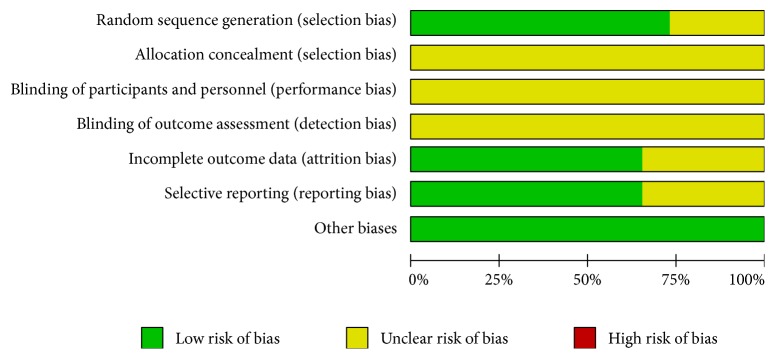
Risk of bias and quality assessment score (%) for studies included in the meta-analysis.

Assessment of study quality is presented in Figure 2. A total of 19 (73.1%) studies were randomized, but the risks of bias due to allocation concealment and blinding were unclear. Sixteen studies had no missing outcomes data. The risk of selective outcomes reporting was unclear in nine studies. Across studies, the risk of bias from other sources was low.

**Figure 3:**
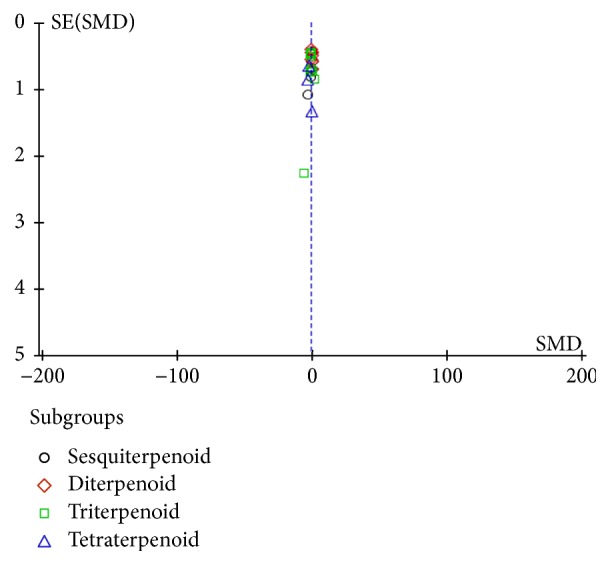
A funnel plot for evaluating publication bias.

Visual inspection of a funnel plot revealed substantial publication bias (Figure 3).

### 3.4. Effect of Terpenoids on Atherosclerotic Lesion Area

**Figure 4:**
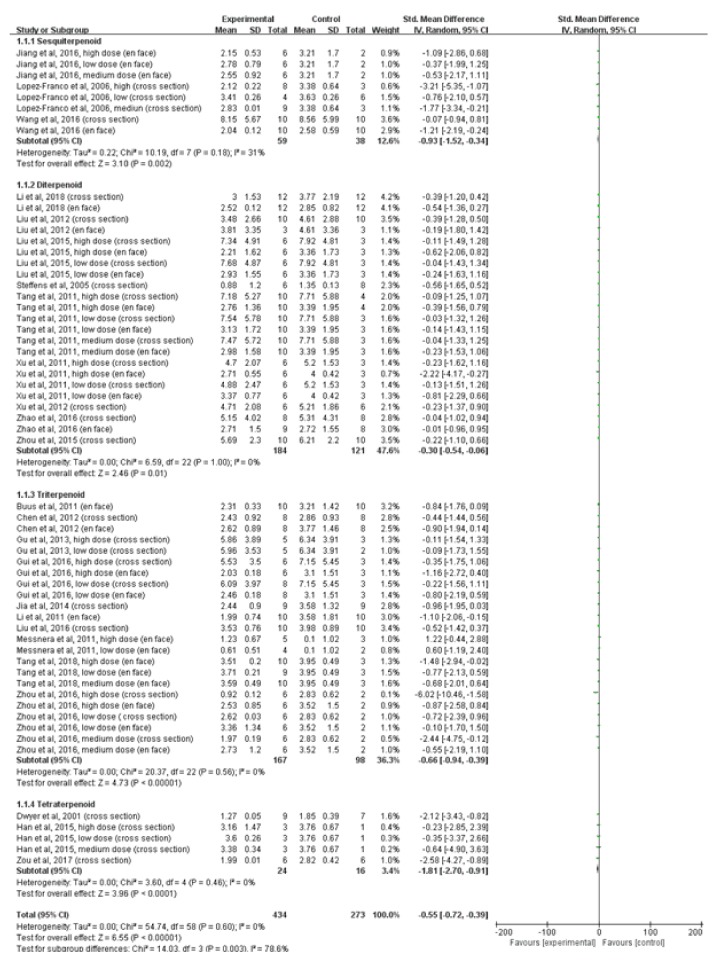
A forest plot of the effects of different terpenoids types on atherosclerotic lesion area. Subgroup analyses evaluated the effects of sesquiterpenoid, diterpenoid, triterpenoid, and tetraterpenoid. SD, standard deviation; CI, confidence interval; Std, standard; IV, inverse variance.

**Figure 5:**
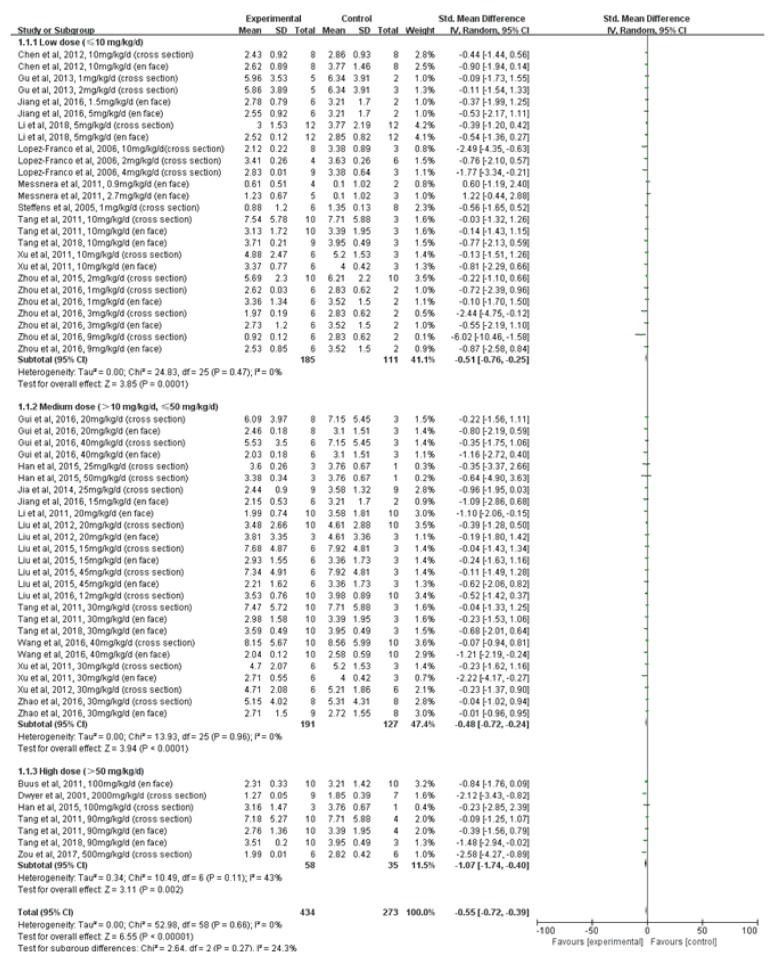
A forest plot of the effects of different terpenoids doses on atherosclerotic lesion area. Subgroup analyses evaluated the effects of low dose (≤10 mg/kg/d), medium dose (>10 mg/kg/d, ≤50 mg/kg/d), and high dose (>50 mg/kg/d). SD, standard deviation; CI, confidence interval; Std, standard; IV, inverse variance.

The effect of terpenoids on atherosclerotic lesion area was reported for 59 datasets obtained from 25 studies (*n*=434, ApoE -/- mice administered terpenoid;* n*=273, ApoE -/- mice administered vehicle control). The meta-analysis demonstrated that overall terpenoids significantly reduced atherosclerotic lesion area when compared to vehicle control (*P*<0.00001; SMD: -0.55; 95% CI: -0.72, -0.39). There was no evidence of heterogeneity between studies (*I*^*2*^=0%, Figures 4 and 5).

Subgroup analyses were conducted to investigate the effects of terpenoid type and dose on atherosclerotic lesion area. In terpenoid type subgroup analyses, sesquiterpenoid (*n*=59, ApoE -/- mice administered sesquiterpenoid;* n*=38, ApoE -/- mice administered vehicle control) significantly reduced atherosclerotic lesion area when compared to vehicle control (*P*=0.002; SMD: -0.93; 95% CI: -1.52, -0.34); there was evidence of moderate heterogeneity between studies (*I*^*2*^=31%). Diterpenoid (*n*=184, ApoE -/- mice administered diterpenoid;* n*=121, ApoE -/- mice administered vehicle control;* P*=0.01; SMD: -0.30; 95% CI: -0.54, -0.06), triterpenoid (*n*=167, ApoE -/- mice administered triterpenoid;* n*=98, ApoE -/- mice administered vehicle control;* P*<0.00001; SMD: -0.66; 95% CI: -0.94, -0.39), and tetraterpenoid (*n*=24, ApoE -/- mice administered tetraterpenoid;* n*=16, ApoE -/- mice administered vehicle control;* P*<0.00001; SMD: -1.81; 95% CI: -2.70, -0.91) significantly reduced atherosclerotic lesion area when compared to vehicle control; there was no evidence of heterogeneity between studies (*I*^*2*^=0%). In terpenoid dose subgroup analyses, high dose group (*n*=58, ApoE -/- mice administered terpenoid >50 mg/kg/d;* n*=35, ApoE -/- mice administered vehicle control) significantly reduced atherosclerotic lesion area when compared to vehicle control (*P*=0.002; SMD: -1.07; 95% CI: -1.74, -0.40); there was evidence of moderate heterogeneity between studies (*I*^*2*^=43%). Low dose group (*n*=185, ApoE -/- mice administered terpenoid ≤10 mg/kg/d;* n*=111, ApoE -/- mice administered vehicle control;* P*=0.0001; SMD: -0.51; 95% CI: -0.76, -0.25) and medium dose group (*n*=191, ApoE -/- mice administered terpenoid >10 mg/kg/d, ≤50 mg/kg/d;* n*=127, ApoE -/- mice administered vehicle control;* P<*0.0001; SMD: -0.48; 95% CI: -0.72, -0.24) significantly reduced atherosclerotic lesion area when compared to vehicle control; there was no evidence of heterogeneity between studies (*I*^*2*^=0%).

Sensitivity analysis that substituted the fixed effect model for the random effects model did not change the overall findings (SMD -0.55 (-0.72, -0.39)* vs.* -0.58 (-0.69, -0.46) and SMD -0.55 (-0.72, -0.39)* vs.* -0.57 (-0.68, -0.45)).

## 4. Discussion

The use of animal models provides a valuable approach to preclinical research, which informs treatment strategies for human diseases. Previous evidence from experiments in animals suggests that natural terpenoids have potential benefits for the treatment of atherosclerosis. However, parameters such as type and age of animal, sample size, housing conditions, and length of follow-up vary across studies. A synthesis and quantitative analysis of the data from animal models that accounts for these sources of heterogeneity may provide insight into the benefits of terpenoids as clinically desirable therapeutic agents in atherosclerosis. Therefore, we performed this systematic review and meta-analysis to evaluate the effects terpenoid administration on atherosclerotic lesion area in ApoE -/- mice. Findings showed that terpenoid administration significantly reduced aortic atherosclerosis lesion area compared to vehicle control.

The terpenoid family constitutes several members, including hemiterpenoid, monoterpenoid, sesquiterpenoid, diterpenoid, sesterterpenoid, triterpenoid, tetraterpenoid, and polyterpenoid [60]. In subanalyses stratified by number of isoprene groups, sesquiterpenoid, diterpenoid, triterpenoid, and tetraterpenoid significantly reduced aortic atherosclerosis lesion area compared to vehicle control in ApoE-/- mice.

To the authors' knowledge, this systematic review and meta-analysis is the first to evaluate the effects of terpenoids on atherosclerosis in ApoE -/- mice. Findings are expected to provide a scientific basis for clinical trials of terpenoids in cardiovascular diseases.

There was no heterogeneity between studies in the overall analysis, but there was a moderate degree of heterogeneity between studies in the analysis of sesquiterpenoid and high dose group. Potential sources of heterogeneity include age and sex of mice and diet administered, each of which can influence the progression of atherosclerosis [1, 61]. Furthermore, method of measurement can affect the assessment. Ten studies reported cross-sectional aortic lesion area, five studies reported longitudinal aortic lesion area, and ten studies reported both cross-sectional and longitudinal aortic lesion area.

*Study Limitations.* This meta-analysis was associated with several limitations. First, the relevance of our findings to humans is limited by species specific differences in lipoprotein metabolism and vascular physiology [62]. The ApoE -/- mouse model is well established for studying atherosclerosis, and the principal characteristics and progression of atherosclerosis in ApoE -/- mice and human subjects appear similar [15, 16]; however, there are differences in pathogenesis. Specifically, the location of the atherosclerotic plaque may differ due to variations in heart rate, blood pressure, and hemodynamics. Atherosclerotic plaque builds up in the root of the aorta and in the brachiocephalic artery in ApoE -/- mice and the coronary artery, the carotid artery, the iliac artery, and the arteries of the lower limb in humans. Second, age is a risk factor in progression of atherosclerosis; therefore, due to differences in life cycle, the natural history of atherosclerotic disease in mice cannot be directly translated to humans. Third, patients usually present to the clinic with advanced atherosclerosis, but the included studies administered terpenoids to mice before disease had progressed and were therefore evaluating early prevention rather than benefit of treatment in advanced disease. Fourth, other animal models of atherosclerosis, such as LDLR -/- mice, rabbits, and hamsters, were not included in this meta-analysis. Fifth, only one parameter, atherosclerotic lesion area, was used to evaluate the effects of terpenoids on atherosclerosis. Other parameters, such as low density lipoprotein (LDL), high density lipoprotein (HDL), total cholesterol (TC), triglycerides (TG), and body mass index (BMI), were not considered. [63] Sixth, the sample sizes of some included studies were relatively small. Seventh, hemiterpenoid, monoterpenoid, and polyterpenoid were not assessed in the analysis. Further investigations in animal models using larger sample sizes are warranted to determine if terpenoids are beneficial for the treatment of atherosclerosis in humans.

The present meta-analysis revealed that terpenoid administration is effective for attenuating aortic atherosclerosis in ApoE-/- mice. In particular, sesquiterpenoid, diterpenoid, triterpenoid, and tetraterpenoid have a potential therapeutic effect with a wide range of doses. Large scale, prospective, and well-designed animal studies are needed to enhance our knowledge of the mechanism of terpenoids for the treatment of atherosclerosis. Randomized controlled trails in humans are required to confirm that terpenoids have clinical benefit as antiatherosclerotic agents.

## Figures and Tables

**Table 1:** Characteristics of the included studies.

Study	Terpenoid	Age	Sex	Diet	Study length	Dose	Route	Location of lesion area	Analysis	Staining	Groups and sample size
Jiang et al., 2016 [19]	Sesquiterpenoid, artesunate	4W	?	HFD	24W	1.5 mg/kg/d 5 mg/kg/d 15 mg/kg/d	i.p.	Thoracic aorta	En face (longitudinal)	Oil red O	Control=6 Sesquiterpenoid low=6 Sesquiterpenoid medium=6 Sesquiterpenoid high=6

Lopez-Franco et al., 2006 [20]	Sesquiterpenoid, parthenolide	12W	Male	HFD	(1) 10W (2) 20W	4 mg/kg/d 10 mg/kg/d 2 mg/kg/d	i.p.	Aortic root	Cross-sectional	Oil red O	Control=6, 6 Sesquiterpenoid low=4 Sesquiterpenoid medium=9 Sesquiterpenoid high=8

Wang et al., 2016 [21]	Sesquiterpenoid, patchouli	8W	Female	HFD	10W	40 mg/kg/d	IG	(1) Thoracic and abdominal aorta (2) Aortic root	(1) En face (longitudinal) (2) Cross-sectional	Oil red O	Control=10, 10 Sesquiterpenoid=10, 10

Li et al., 2018 [22]	Diterpenoid, pseudolaric acid B	8W	Male	HFD	4W	5 mg/kg/d	IG	(1) Thoracic and abdominal aorta (2) Aortic root	(1) En face (longitudinal) (2) Cross-sectional	Oil red O	Control=12, 12 Diterpenoid=12, 12

Liu et al., 2012 [23]	Diterpenoid, ginkgolide B	8W	Male	HFD	8W	20 mg/kg/d	IG	(1) Thoracic aorta (2) Aortic sinus	(1) En face (longitudinal) (2) Cross-sectional	Oil red O	Control=3, 10 Diterpenoid treatment=3, 10

Liu et al., 2015 [24]	Diterpenoid, cryptotanshinone	6W	Male	HFD	16W	15 mg/kg/d 45 mg/kg/d	IG	(1) Aortic root to iliac branches (2) Aortic sinus	(1) En face (longitudinal) (2) Cross-sectional	Oil red O	Control=6, 6 Diterpenoid low=6, 6 Diterpenoid high=6, 6

Steffens et al., 2005 [25]	Diterpenoid, delta-9-tetrahydrocannabinol	10W	Male	HFD	6W	1 mg/kg/d	IG	Aortic root	Cross-sectional	Sudan IV	Control=8 Diterpenoid treatment=6

Tang et al., 2011 [26]	Diterpenoid, tanshinone IIA	6W	Male	NCD	20W	10 mg/kg/d 30 mg/kg/d 90 mg/kg/d	IG	(1) Aortic arch (2) Aortic root	(1) En face (longitudinal) (2) Cross sectional	(1) Oil red O (2) HE	Control=10, 10 Diterpenoid low=10, 10 Diterpenoid medium=10, 10 Diterpenoid high=10, 10

Xu et al., 2011 [27]	Diterpenoid, tanshinone IIA	6W	Male	HCD	16W	10 mg/kg/d 30 mg/kg/d	IG	(1) Aortic root to iliac branches (2) Aortic sinus	(1) En face (longitudinal) (2) Cross-sectional	Oil red O	Control=6, 6 Diterpenoid low=6, 6 Diterpenoid high=6, 6

Xu et al., 2012 [28]	Diterpenoid, tanshinone IIA	6W	Male	HCD	16W	30 mg/kg/d	IG	Aortic roots	Cross-sectional	Oil red O	Control=6 Diterpenoid treatment=6

Zhao et al., 2016 [29]	Diterpenoid, tanshinone IIA	8W	Male	HFD	8W	30 mg/kg/d	IG	Thoracic aorta	(1) En face (longitudinal) (2) Cross-sectional	Oil red O	Control=8, 8 Diterpenoid treatment=9, 8

Zhou et al., 2015 [30]	Diterpenoid, retinoic acid	8W	Male	HFD	8W	2 mg/kg/d	i.p.	Aortic sinus	Cross-sectional	Oil red O	Control=10 Diterpenoid treatment=10

Buus et al., 2011 [31]	Triterpenoid, oleanolic acid	12W	Male	HFD	8W	100 mg/kg/d	IG	Aortic root to thoracic aorta	En face (longitudinal)	Oil red O	Control=10 Triterpenoid =10

Chen et al., 2012 [32]	Triterpenoid, corosolic acid	8W	Male	HFD	12W	10 mg/kg/d	Chow	(1) Aortic root to abdominal aorta (2) Aortic root	(1) En face (longitudinal) (2) Cross-sectional	(1) Sudan IV (2) HE	Control=8, 8 Triterpenoid=8, 8

Gu et al., 2013 [33]	Triterpenoid, celastrol	8W	Male	HFD	4W	1 mg/kg/d 2 mg/kg/d	i.p.	Aortic sinus	Cross-sectional	Oil red O	Control=5 Triterpenoid low=5 Triterpenoid high=5

Gui et al., 2016 [34]	Triterpenoid, betulin	6W	Male	HFD	12W	20 mg/kg/d 40 mg/kg/d	Chow	(1) Aortic root to iliac branches (2) Aortic sinus	(1) En face (longitudinal) (2) Cross-sectional	(1) Sudan IV (2) Oil red O	Control=6, 6 Triterpenoid low=8, 8 Triterpenoid high=6, 6

Jia et al., 2014 [35]	Triterpenoid, notoginsenoside R1	9W	Male	HFD	8W	25 mg/kg/d	i.p.	Aortic root	Cross-sectional	HE	Control=9 Triterpenoid treatment=9

Li et al., 2011 [36]	Triterpenoid, ginsenoside-Rd	6W	?	HFD	12W	20 mg/kg/d	i.p.	Aortic root to iliac branches	En face (longitudinal)	Oil red O	Control=10 Triterpenoid=10

Liu et al., 2016 [37]	Triterpenoid, ilexgenin A	8W	Male	HFD	16W	12 mg/kg/d	Chow	Aortic sinus	Cross-sectional	HE	Control=10 Triterpenoid =10

Messner et al., 2011 [38]	Triterpenoid, ursolic acid	8W	Male	HFD	24W	0.9 mg/kg/d 2.7 mg/kg/d	Drink	Aortic root to iliac branches	En face (longitudinal)	Sudan IV	Control=5 Triterpenoid low=4 Triterpenoid high=5

Tang et al., 2018 [39]	Triterpenoid, celosins	12W	Male	HFD	4W	10 mg/kg/d 30 mg/kg/d 90 mg/kg/d	Chow	Aortic root to iliac branches	En face (longitudinal)	Oil red O	Control=9 Triterpenoid low=9 Triterpenoid medium=10 Triterpenoid high=10

Zhou et al., 2016 [40]	Triterpenoid, compound K	10W	Male	HFD	8W	1 mg/kg/d 3 mg/kg/d 9 mg/kg/d	i.p.	(1) Aortic root to abdominal aorta (2) Aortic root	(1) En face (longitudinal) (2) Cross-sectional	(1) Oil red O (2) HE	Control=6, 6 Triterpenoid low=6, 6 Triterpenoid medium=6, 6 Triterpenoid high=6, 6

Dwyer et al., 2001 [41]	Tetraterpenoid, lutein	12W	Female	NCD	8W	2000 mg/kg/d	Chow	Aortic root	Cross-sectional	Oil red O	Control=7 Tetraterpenoid=9

Han et al., 2015 [42]	Tetraterpenoid, lutein	8W	Male	HFD	24W	25 mg/kg/d 50 mg/kg/d 100 mg/kg/d	Chow	Aortic sinus	Cross-sectional	Oil red O	Control=3 Tetraterpenoid low=3 Tetraterpenoid medium=3 Tetraterpenoid high=3

Zou et al., 2017 [43]	Tetraterpenoid, astaxanthin	6W	Male	NCD	12W	500 mg/kg/d	Chow	Aortic sinus	Cross-sectional	Oil red O	Control=6 Tetraterpenoid treatment=6

Note: NCD, normal-chow diet; HCD, high-cholesterol diet; HFD, high-fat diet; IG, intragastric; i.p., intraperitoneal injection; HE, hematoxylin and eosin; ? = not reported.

## Data Availability

The data used and/or analyzed during the current study are available from the corresponding author on reasonable request.
